# Cytocam-IDF (incident dark field illumination) imaging for bedside monitoring of the microcirculation

**DOI:** 10.1186/s40635-015-0040-7

**Published:** 2015-01-31

**Authors:** Guclu Aykut, Gerke Veenstra, Claudia Scorcella, Can Ince, Christiaan Boerma

**Affiliations:** Department of Intensive Care, Erasmus MC University Medical Center, Dr. Molewaterplein 50, Rotterdam, 3015 GE The Netherlands; Department of Intensive Care, Medical Centre Leeuwarden, PO Box 888, 8901BR Leeuwarden, The Netherlands; Department of Anesthesiology, University Hospital Heidelberg, Im Neuenheimer Feld 110, 69120 Heidelberg, Germany

**Keywords:** Microcirculation, SDF imaging, Cytocam-IDF imaging, İntravital microscopy, Sepsis

## Abstract

**Background:**

Orthogonal polarized spectral (OPS) and sidestream dark field (SDF) imaging video microscope devices were introduced for observation of the microcirculation but, due to technical limitations, have remained as research tools. Recently, a novel handheld microscope based on incident dark field illumination (IDF) has been introduced for clinical use. The Cytocam-IDF imaging device consists of a pen-like probe incorporating IDF illumination with a set of high-resolution lenses projecting images on to a computer controlled image sensor synchronized with very short pulsed illumination light. This study was performed to validate Cytocam-IDF imaging by comparison to SDF imaging in volunteers.

**Methods:**

This study is a prospective, observational study. The subjects consist of 25 volunteers.

**Results:**

Sublingual microcirculation was evaluated using both techniques. The main result was that Cytocam-IDF imaging provided better quality images and was able to detect 30% more capillaries than SDF imaging (total vessels density Cytocam-IDF: 21.60 ± 4.30 mm/mm^2^ vs SDF: 16.35 ± 2.78 mm/mm^2^, *p* < 0.0001). Comparison of the images showed increased contrast, sharpness, and image quality of both venules and capillaries.

**Conclusions:**

Cytocam-IDF imaging detected more capillaries and provided better image quality than SDF imaging. It is concluded that Cytocam-IDF imaging may provide a new improved imaging modality for clinical assessment of microcirculatory alterations.

**Electronic supplementary material:**

The online version of this article (doi:10.1186/s40635-015-0040-7) contains supplementary material, which is available to authorized users.

## Background

Microcirculation is the main means of oxygen delivery to tissue cells and is essential for the maintenance of cellular life and function. Its function relies on the complex interaction of its component cellular systems, including red and white blood cells, endothelial, smooth muscle, and parenchymal cells. The function of the organs is directly dependent on the function of their respective microcirculation, and achievement of good microcirculatory function can be considered to be the prime goal of the cardiovascular system and of particular importance to critically ill patients, especially ones who are in shock [1]. Many studies have demonstrated that persistent microcirculation alterations that are unresponsive to therapy are independently associated with adverse outcome, especially in septic patients [1-5]. Additionally, these microcirculatory alterations have been shown in various studies to be independent of systemic hemodynamic variables, making the observation of microcirculation a potentially important extension of the conventional systemic hemodynamic monitoring of critically ill patients [3,4].

In the early 20th century, direct intravital observation of human microcirculation was limited to the use of bulky capillary microscopes, which were mainly applied to the nailfold capillary bed. In 1964, Krahl made use of incident light directed at an oblique angle to the study tissue surfaces [6]. In 1971, Sherman et al. introduced a new method of microcirculation observations called incident dark field illumination (IDF) microscopy. This method enabled observations of organ surface microcirculation using epi-illumination, without the need for transillumination of the tissue from below [7]. An alternative method to observe microcirculation using epi-illumination was introduced by Slaaf et al., enabling the imaging of subsurfaces using cross polarized light microscopy [8]. In the late 1990s, Groner et al. adapted the Slaaf et al. technique to a handheld video microscope [9]. This method was called orthogonal polarization spectral (OPS) imaging. We validated and introduced this technique to patients and were able for the first time to produce organ surface microcirculation images in surgical patients [10,11]. This technique opened up the field of studying human microcirculation in organ and tissue surfaces at the bedside especially in critically ill patients.

OPS imaging can be regarded as the first generation handheld bedside imaging instrument to be applied to critically ill patients, resulting in general recognition that microcirculation is an important physiological process that is compromised during critical illness and needs to be monitored in a clinical environment [12,13]. OPS imaging was improved upon by our development of a second generation handheld analogue video microscopes based on sidestream dark field (SDF) imaging [14]. Its advancement was that it provided better images than OPS imaging and allowed battery operation, making the device more mobile than its predecessor. A device similar to SDF imaging device fitted with a USB extension called the Capiscope was also recently introduced [15]. These devices, however, remained research tools mainly due to the technological limitations preventing operator independent reproducible measurements and the inability to achieve automatic microcirculation analysis for quantification needed for clinical decision making [16-18]. Analysis of the images to extract relevant functional microcirculatory parameters required time-consuming off-line analysis [16] precluding their use in bedside clinical decision making and in titrating therapy to reach microcirculatory end points [19].

Cytocam-IDF imaging can be regarded as third generation handheld microscope because it employs a completely new hardware platform where a high density pixel-based imaging chip and short pulsed illumination source under computer control synchronizes and controls illumination and image acquisition. The device consists of a pen-like probe incorporating IDF illumination with a set of high-resolution lenses projecting images on to a computer controlled high-density image sensor synchronized to an illumination unit. The probe is covered by a sterilizable cap. Cytocam-IDF imaging is based on the IDF, a principle originally introduced by Sherman and Cook [7]. It further incorporates a stepping motor for quantitative focusing as well as high-resolution optics.

In the first part of this study, Cytocam-IDF imaging is validated by quantitative comparison of microcirculatory parameters to SDF imaging in sublingual tissue using specialized image processing software developed earlier by us [20]. In addition, Cytocam-IDF and SDF images of one and the same sublingual microcirculatory area were obtained to directly compare the image quality to each other in the second part of the study. This feature allows serial measurements to be made without the need to refocus, an important feature with respect to previous generation devices which require time-consuming manual adjustment of focus dials.

### Subjects

Twenty-five healthy volunteers (8 males and 17 females) between the ages 23 and 55 were recruited. None of the subjects had history or evidence of disease or were taking drugs that are known to affect microcirculatory function.

## Methods

### SDF imaging

In SDF imaging (Microscan, MicroVision Medical, Amsterdam, The Netherlands), illumination is provided by surrounding a central light guide with concentrically placed light-emitting diodes (LEDs) to provide sidestream dark field illumination [14]. The magnification lens in the core of the light guide is optically isolated from the illuminating outer ring, thus preventing tissue surface reflections. Light from the illuminating outer core of the SDF probe penetrates the tissue and illuminates the tissue-embedded microcirculation by scattering. The LEDs use green light (530 nm wavelength) corresponding to an isobestic point in the absorption spectra of oxyhemoglobin and de-oxyhemoglobin. The LEDs provide pulsed illumination to overcome the interlacing of the analogue video cameras used. The SDF device with a total weight of 320 g is fitted with a 5× objective lens. It is based on an analogue video camera which allows its output to be directly connected to a television monitor. For analysis of the video sequences, images need to be digitized using an external analogue to digital converting device and then analyzed off-line using specialized image-processing software [20]. Illumination intensity and image focus are adjusted manually by a dial on the devices. The probe, covered by a sterile disposable cap, can be placed on organ and tissue surfaces to observe the microcirculation.

### Cytocam-IDF imaging

**Figure 1:**
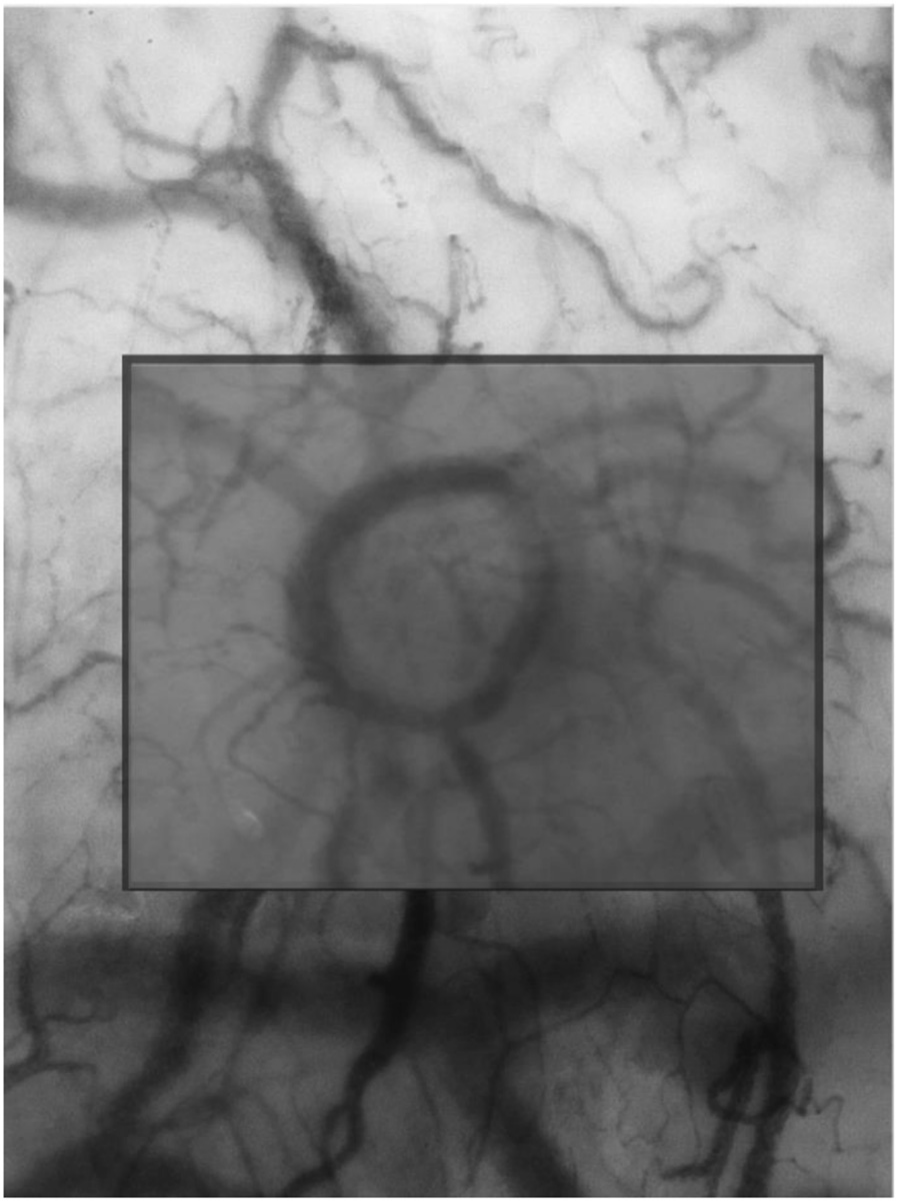
**Smaller SDF image in larger Cytocam-IDF image.** This figure shows the field of view of SDF and Cytocam-IDF imaging superimposed on each other showing the larger field of view offered by the larger image sensor used by the later technique.

Cytocam-IDF imaging (Braedius Medical, Huizen, The Netherlands) consists of a combination of IDF illumination with optical and technical features optimized for visualization of the microcirculation on organ surfaces. It uses incident dark field illumination [7] with high-brightness LEDs with a very short illumination pulse time of 2 ms. The image acquisition and sensor are under computer control and electronically synchronized to the illumination pulses. This feature, in combination with a specialized set of lenses, projects images onto a computer controlled image sensor and results in high penetration sharp contour visualization of the microcirculation showing flowing red and white blood cells. The device is constructed of aluminum and titanium, resulting in a lightweight (120 g) and pen-like instrument (length 220 mm, diameter 23 mm). The camera is fully digital with a high-resolution sensor, which is used in binning mode, resulting in a 3.5 megapixel frame size. The combination of an optical magnification factor of 4 and the large image area of the sensor provides a field of view of 1.55 × 1.16 mm about three times larger than the field of view of previous devices (see Figure 1).

**Figure 2:**
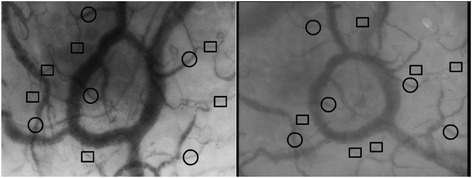
**Histogram points taken for analyses; square capillary; circle venule; left Cytocam-IDF imaging; right SDF-imaging.**

**Figure 3:**
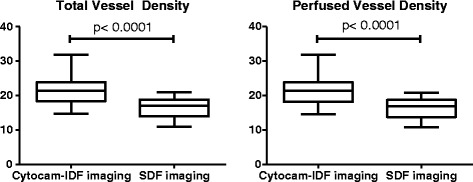
**Boxplots of TVD and PVD.**

The optical system provides an optical resolution of more than 300 lines/mm.

The camera is connected to a device controller based on a powerful medical grade computer that is used for image storage and analysis. The device controller includes a camera adapter with a dedicated microprocessor for controlling the camera. Additionally, the camera adapter enables high-speed data transfer between the camera and controller. The Cytocam-IDF imaging device is supplied with an analysis application for quantification of microcirculatory parameters. The digitally recorded images can be analyzed automatically. It is also possible to analyze the recorded files off-line, as we did for this study. A novel feature of the device is its quantitative focusing mechanism, using a piezo linear motor with an integrated distance measuring system to position the sensor within 2 μm. Investigation has shown that each person has a characteristic depth of focus, which allows serial measurements to be made by pre-setting the characteristic focused depth [21].

### Protocol

#### Comparison of microcirculatory parameters

The volunteers were evaluated in a supine, 30° head-up position. Room temperature was kept between 19°C and 22°C. Demographic data (age, gender, weight, and length), blood pressure, and heart rate were recorded. Blood pressure was measured noninvasively, and heart rate was recorded by plethysmography. The microvascular measurements were obtained as a single measurement in the sublingual mucosa in three different areas with SDF and Cytocam-IDF imaging without special preparation of the mouth. The probe was handheld and adjusted by experienced operators (GA and GV) to obtain optimal image quality. With the SDF technique, after adequate focus and contrast adjustment, steady images of at least 15 s were acquired and recorded on a digital videotape (Sony Video Walkman GV-D 1000E; Sony, Tokyo, Japan), which digitizes the analogue SDF images prior to video storage. The images were captured in representative AVI format video clips (SonyDVgate; Sony, Tokyo, Japan) to allow off-line computerized image analysis using specialized software we had previously developed for this purpose [20].

To use the Cytocam-IDF imaging device, the optimal focus depth and contrast were first adjusted. Steady images of 6 s were acquired and computer stored. The image clips were exported for analysis using the same image analysis software used for the SDF images [20]. Analysis took into account the different magnification of the images by the two different techniques (4× magnification in Cytocam-IDF imaging versus 5× magnification in SDF imaging.

### Analysis

#### Comparison of microcirculatory parameters

The perfusion of a tissue depends on the number, distribution, and diameters of the capillaries in combination with blood viscosity and driving pressure across the capillaries. There are two main hemodynamic principles governing how oxygen in red blood cells reaches the tissue cells; the first is the convection based on red blood cell flow, and the second is the diffusion distance oxygen must travel from the red blood cells in the capillaries to the parenchymal cells [19]. Convection is quantified by measurement of flow in the microvessels, and diffusion is quantified by the density of the perfused microvessels (functional capillary density).

Subsequent image analysis was performed using microvascular density (total or perfused vessel density) and microvascular perfusion (proportion of perfused vessels and microcirculatory flow index) parameters in line with international consensus [22]. Software assisted analysis (AVA 3.0; Automated Vascular Analysis, Academic Medical Center, University of Amsterdam) was used on the images [20]. The analysis of the microvascular density was restricted to vessels with a diameter <20 μm.

The total vessel density (TVD; mm/mm^2^) was determined using the AVA software. A semiquantitative analysis previously validated [23] but assisted by the AVA software was performed in individual vessels that distinguished among no flow (0), intermittent flow (1), sluggish flow (2), and continuous flow (3). A value was assigned for each vessel. The overall score, called the microvascular flow index (MFI), is the average of the individual values [24]. The proportion of perfused vessels (PPV) was calculated as the number of vessels with flow values of 2 and 3 divided by the total number of vessels. Perfused vessel density (PVD) was determined as the total vessel density multiplied by the fraction of perfused vessels [22]. Analyses of all images were done off-line and blinded to the investigators.

#### Sublingual microcirculatory image contrast and sharpness analysis

In the same way we had compared OPS imaging to SDF imaging [14], we evaluated image contrast and sharpness using image analysis software (ImageJ; developed at the US National Institutes of Health, www.nih.gov). Five venules and six capillaries found in one sublingual location, measured sublingually by the two cameras; the capillary and venular contrast, sharpness, and quality were calculated. To determine capillary contrast with respect to the surrounding tissue, cross-sectional grayscale histograms (grayscale value 0 corresponds to black, and 255 corresponds to white) were obtained. The lowest gray value in the capillaries (I min) and the highest gray value in the tissue left (I max, left) and right (I max, right) of the capillaries were measured. The increase of the maximum slope angles *α* left and *α* right of the slopes of the gray value at the capillary-tissue interfaces was calculated. Histogram points taken for analyses are presented in Figure 2.

### Statistical analysis

Statistical analyses were performed using SPSS statistical software, version 21 (version 18/21, SPSS Inc., USA). The Kolmogorov-Smirnov test was used to test whether the data were distributed normally. After identifying a normal distribution, the density parameters were compared by Student's *t* test. As the perfusion parameters did not show a normal distribution, a nonparametric test (Mann-Whitney *U*) was used to compare these parameters. Data are presented as the mean and standard deviation unless otherwise specified. A *p* value <0.05 was considered statistically significant.

## Results

Baseline characteristics are presented in Table 1. Tests for normality showed that TVD and PVD had normal distribution. The vascular density parameters TVD and PVD were significantly higher with Cytocam-IDF imaging than with SDF imaging (TVD-Cytocam-IDF: 21.60 mm/mm^2^ ± 4.30 mm/mm^2^ vs TVD-SDF: 16.35 mm/mm^2^ ± 2.78 mm/mm^2^, *p* < 0.0001 and PVD-Cytocam-IDF: 21.50 mm/mm^2^ ± 4,38 mm/mm^2^ vs PVD-SDF: 16.24 mm/mm^2^ ± 2.81 mm/mm^2^, *p* < 0.0001). Boxplots are presented in Figure 3.

**Table 1:** **Baseline characteristics**

**Variable**	**Results**
Age [years]	33 [27.5 to 46.0]
Gender, male [*n*]	8
Systolic blood pressure [mmHg]	130 [119 to 141]
Diastolic blood pressure [mmHg]	74 [66 to 87]
Mean arterial blood pressure [mmHg]	92 [84 to 102]
Heartrate [beats per minute]	70 [65 to 77]
Weight [kg]	73 [63 to 78]
Height [cm]	173 [170 to 180]

The perfusion parameters MFI and PPV did not differ significantly between the two techniques (Table 2), and the Bland-Altman plot showed no clinically significant bias. Bland-Altman plots are included in the Additional file 1.

**Table 2:** **Microcirculatory parameters**

	**Cytocam**	**SDF**	***p***
MFI small	3.0 [3.0 to 3.0]	3.0 [2.96 to 3.00]	0.289
MFI large	3.0 [3.0 to 3.0]	3.0 [2.96 to 3.00]	0.494
TVD [mm/mm^2^]	21.60 ± 4.30	16.35 ± 2,78	<0.0001*
PPV [%]	100 [99 to 100]	99 [99 to 100]	0.368
PVD [mm/mm^2^]	21.50 ± 4.38	16.24 ± 2.81	<0.0001*

MFI small: <20 um; MFI large: >20 um.

*p < 0.05 is the cutoff value for statistical significance.

### Sublingual microcirculatory image contrast and sharpness analysis

Cytocam-IDF image quality from the sublingual area is significantly better in the capillaries and venules than the SDF image quality (Table 3). The quality score was obtained based on contrast and sharpness, both of which are significantly improved with the Cytocam-IDF imaging in both capillaries and venules.

**Table 3:** **Results from contrast and sharpness analysis**

	**Modality**	**Contrast**	**Sharpness**	**Quality**
Capillaries	SDF	0.04 ± 0.01	0.78 ± 0.06	0.03 ± 0.01
	Cytocam	0.99 ± 0.03	0.90 ± 0.05	0.09 ± 0.03
		*p* = 0.004*	*p* = 0.003*	*p* = 0.005*
Venules	SDF	0.07 ± 0.03	0.82 ± 0.07	0.06 ± 0.03
	Cytocam	0.12 ± 0.03	0.88 ± 0.05	0,11 ± 0.03
		*p* = 0.037*	*p* = 0.144*	*p* = 0.037*

*p < 0.05 is the cutoff value for statistical significance.

## Discussion

In this study, we validated Cytocam-IDF imaging, a third generation novel lightweight computer-controlled imaging sensor-based handheld microscope, by comparing it to a second generation device, SDF imaging. Our results showed that Cytocam-IDF imaging visualized more (30%) microvessels as quantified by measurement of total vascular density parameters in the sublingual microcirculation than did SDF imaging. In addition, our study showed that Cytocam-IDF imaging provided improved image quality with respect to SDF imaging in terms of contrast and image sharpness. Similar results were found in a recent different preliminary validation study comparing Cytocam-IDF imaging to SDF imaging in neonates [25].

It is likely that the significantly higher vascular density, as observed with the new Cytocam-IDF technique in comparison to SDF imaging, is the direct result of the observed increase in contrast and sharpness, due to the improved magnification lens and high-resolution sensor in combination with more precise quantitative focusing. Furthermore, since the new device is fully digitalized, there is no loss of image quality in the conversion process from analogue to digital. Analogue cameras have the disadvantage of alternatively scanning odd and even video lines, resulting in a loss of resolution in the time domain as is the case in the SDF camera. An alternative explanation could be the reduction in pressure artefacts in the lighter Cytocam-IDF device in comparison to the much heavier SDF device resulting in compressed microvessels becoming now more visible. We think, however, that this is unlikely since pressure artefacts are mainly reflected in a reduction in red blood cell velocity characteristically in the larger, venular, vessels. Since this study was performed in healthy individuals, such flow abnormalities should be absent, as is the case in the observations in both systems. Therefore, it seems likely that the observed difference in capillary density is not related to reduction pressure artefacts.

This conclusion is in line with the second important observation of the study, the absence of difference in variables of red blood cell velocity, such as MFI and PPV. Previous publications report a ‘normal’ MFI in healthy volunteers, equal or close to 3, and a PPV close to 100% [4,5,26,27]. Therefore, the absence of difference in MFI and PPV between the two methods can be considered as an important quality check of the observations found in the present study.

Although the visualization of vessels with the new device is based on the same physical principal of indirect background illumination applied in all such devices, there are clearly new features with relevance to the development of research in this field. The high-resolution sensor combined with lenses especially made for microcirculatory imaging makes the optical resolution higher than the SDF system. As a result, more capillaries become visible (Figure 2), with substantial implications for the observation of the microcirculation in several disease states. This also has the potential to observe smaller structures such as vessel wall abnormalities and possibly the endothelial glycocalyx. The new quantitative focusing mechanism not only allows more precise focusing but also maintains optimal focusing depth throughout a measurement allowing multiple observations to be made over time without the need to refocus each time a measurement is made. Focus of ongoing research, i.e., measurements for extended periods of time on one and the same spot, is currently not possible, especially in the nonsedated patients. As such, the potential to maintain optimal focusing depth reduces the variability in observations. The potential for development of treatment based on the on-line analysis of the fully digitalized images and increase in frame rate are outside the scope of this article.

Two studies [4,27] have reported sublingual vascular density values in mm/mm^2^ in volunteers using SDF imaging. Ours are in exact agreement with the values found by Edul et al. who used similar AVA software. However, SDF-derived TVD in our experiment is lower than in comparison to those found by Hubble et al., but this may be explained by a difference in software analysis (Capiscope, KK Technology, Axminster, UK). Agreement was found however between the three studies on the finding that in volunteers almost all vessels exhibit flow.

Clearly, the results of this study are limited to healthy volunteers, and further validation is needed in the clinical setting. However, heterogeneity of blood flow within the catchment area of the device has now been recognized as a key characteristic in many disease states [5]. To this end, the consensus paper decided to obtain three to five video clips per observation and report the average of the variables [28]. It was key to our experiment to exclude this heterogeneity.

Secondly, our data do not deal with the potential influence of intra-observer variability. Although a substantial variability has been reported in the jejunal mucosal microcirculation of pigs [29], multiple studies have confirmed the excellent reproducibility in the human sublingual area [4,5,26].

## Conclusions

In this study, we validated a third generation novel lightweight computer-controlled imaging sensor-based handheld microscope called the Cytocam-IDF imaging by comparing it to a second generation device, SDF imaging. Our results showed that Cytocam-IDF imaging was able to detect more capillaries in terms of density and provided improved quality image in the sublingual microcirculation. Considering the improved image quality along with its light weight and ability to automatically analyze images, we expect it to contribute to the clinical assessment of microcirculation alterations in various clinical scenarios.

## Additional file

Additional file 1:
**Bland-Altman MFI and PPV.** Due to substantial overlap, multiple observations may be represented as one dot.

## References

[1] De Backer D, Donadello K, Sakr Y, Ospina-Tascon G, Salgado D, Scolletta S (2013). Microcirculatory alterations in patients with severe sepsis: impact of time of assessment and relationship with outcome. Crit Care Med.

[2] Ince C (2005). The microcirculation is the motor of sepsis. Crit Care.

[3] Top APC, Ince C, de Meij N, van Dijk M, Tibboel D (2011). Persistent low microcirculatory vessel density in nonsurvivors of sepsis in pediatric intensive care. Crit Care Med.

[4] Edul VSK, Enrico C, Laviolle B, Vazquez AR, Ince C, Dubin A (2012). Quantitative assessment of the microcirculation in healthy volunteers and in patients with septic shock. Crit Care Med.

[5] Trzeciak S, Dellinger RP, Parrillo JE, Guglielmi M, Bajaj J, Abate NL (2007). Early microcirculatory perfusion derangements in patients with severe sepsis and septic shock: relationship to hemodynamics, oxygen transport, and survival. Ann Emerg Med.

[6] Krahl VE (1962). Observations on the pulmonary alveolus and its capillary circulation in the living rabbit. Anat Rec.

[7] Sherman H, Klausner S, Cook WA (1971). Incident dark-field illumination: a new method for microcirculatory study. Angiology.

[8] Slaaf DW, Tangelder GJ, Reneman RS, Jäger K, Bollinger A (1987). A versatile incident illuminator for intravital microscopy. Int J Microcirc Clin Exp.

[9] Groner W, Winkelman JW, Harris AG, Ince C, Bouma GJ, Messmer K (1999). Orthogonal polarization spectral imaging: a new method for study of the microcirculation. Nat Med.

[10] Mathura KR, Vollebregt KC, Boer K, De Graaff JC, Ubbink DT, Ince C (2001). Comparison of OPS imaging and conventional capillary microscopy to study the human microcirculation. J Appl Physiol.

[11] Mathura KR, Bouma GJ, Ince C (2001). Abnormal microcirculation in brain tumours during surgery. Lancet.

[12] De Backer D, Creteur J, Preiser JC, Dubois MJ, Vincent JL (2002). Microvascular blood flow is altered in patients with sepsis. Am J Respir Crit Care Med.

[13] Spronk PE, Ince C, Gardien MJ, Mathura KR, Oudemans-van Straaten HM, Zandstra DF (2002). Nitroglycerin in septic shock after intravascular volume resuscitation. Lancet.

[14] Goedhart PT, Khalilzada M, Bezemer R, Merza J, Ince C (2007). Sidestream dark field (SDF) imaging: a novel stroboscopic LED ring-based imaging modality for clinical assessment of the microcirculation. Opt Express.

[15] Dababneh L, Cikach F, Alkukhun L, Dweik RA, Tonelli AR (2014). Sublingual microcirculation in pulmonary arterial hypertension. Ann Am Thoracic Soc.

[16] Mik EG, Johannes T, Fries M (2009). Clinical microvascular monitoring: a bright future without a future?. Crit Care Med.

[17] Sallisalmi M, Oksala N, Pettilä V, Tenhunen J (2012). Evaluation of sublingual microcirculatory blood flow in the critically ill. Acta Anaesthesiol Scand.

[18] Bezemer R, Bartels SA, Bakker J, Ince C (2012). Clinical review: clinical imaging of the sublingual microcirculation in the critically ill - where do we stand?. Crit Care.

[19] Ince C (2014). The rationale for microcirculatory guided fluid therapy. Curr Opin Crit Care.

[20] Dobbe JGG, Streekstra GJ, Atasever B, van Zijderveld R, Ince C (2008). Measurement of functional microcirculatory geometry and velocity distributions using automated image analysis. Med Biol Eng Comput.

[21] Milstein D, Romay E, Ince C (2012). A novel computer-controlled high resolution video microscopy imaging system enables measuring mucosal subsurface focal depth for rapid acquisition of oral microcirculation video images. Intensive Care Med.

[22] De Backer D, Hollenberg S, Boerma C, Goedhart P, Büchele G, Ospina-Tascon G (2007). How to evaluate the microcirculation: report of a round table conference. Crit Care.

[23] Dubin A, Pozo MO, Ferrara G, Murias G, Martins E, Canullán C (2009). Systemic and microcirculatory responses to progressive hemorrhage. Intensive Care Med.

[24] Boerma EC, Mathura KR, van der Voort PHJ, Spronk PE, Ince C (2005). Quantifying bedside-derived imaging of microcirculatory abnormalities in septic patients: a prospective validation study. Crit Care.

[25] van Elteren H, van den Berg V, de Jonge R, Ince C, Reiss I (2014). Cutaneous microcirculation in preterm neonates: comparison between sidestream darkfield (SDF) and incident darkfield (IDF) imaging. Pediatr Crit Care Med.

[26] De Backer D, Creteur J, Dubois MJ, Sakr Y, Vincent JL (2004). Microvascular alterations in patients with acute severe heart failure and cardiogenic shock. Am Heart J.

[27] Hubble SMA, Kyte HL, Gooding K, Shore AC (2009). Variability in sublingual microvessel density and flow measurements in healthy volunteers. Microcirculation.

[28] De Backer D, Hollenberg S, Boerma C, Goedhart P, Büchele G, Ospina-Tascon G (2007). How to evaluate the microcirculation: report of a round table conference. Crit Care.

[29] Bracht H, Krejci V, Hiltebrand L, Brandt S, Sigurdsson G, Ali SZ (2008). Orthogonal polarization spectroscopy to detect mesenteric hypoperfusion. Intensive Care Med.

