# Supplementary material for: Cytocam-IDF (incident dark field illumination) imaging for bedside monitoring of the microcirculation
Source: Intensive Care Med Exp. 2015 Jan 31;3:4. doi: 10.1186/s40635-015-0040-7 (PMC4512989; doi:10.1186/s40635-015-0040-7)
Supplement: Additional file 1: — Bland-Altman MFI and PPV. Due to substantial overlap, multiple observations may be represented as one dot. [file 40635_2015_40_MOESM1_ESM.docx]

Appendix: Bland-Altman MFI and PPV. Due to substantial overlap multiple observations may be represented as one dot.
